# Changes and Appointments

**Published:** 1882-02

**Authors:** 


CHANGES AND APPOINTMENTS.—Dr. Louis Elsberg, of New York, has resigned the position he formerly held in the Medical Department of the New York University, and has received the appointment of Professor of Laryngology and Diseases of the Throat in the Dartmouth Medical College.

